# Exploring the pathogenesis of pulmonary vascular disease

**DOI:** 10.3389/fmed.2024.1402639

**Published:** 2024-07-10

**Authors:** Chidinma Ejikeme, Zeenat Safdar

**Affiliations:** Department of Pulmonary-Critical Care Medicine, Houston Methodist Lung Center, Houston Methodist Hospital, Houston, TX, United States

**Keywords:** pulmonary arterial hypertension, pathogenesis, epigenetic modifications, DNA methylation, histone modification, non-coding RNA

## Abstract

Pulmonary hypertension (PH) is a complex cardiopulmonary disorder impacting the lung vasculature, resulting in increased pulmonary vascular resistance that leads to right ventricular dysfunction. Pulmonary hypertension comprises of 5 groups (PH group 1 to 5) where group 1 pulmonary arterial hypertension (PAH), results from alterations that directly affect the pulmonary arteries. Although PAH has a complex pathophysiology that is not completely understood, it is known to be a multifactorial disease that results from a combination of genetic, epigenetic and environmental factors, leading to a varied range of symptoms in PAH patients. PAH does not have a cure, its incidence and prevalence continue to increase every year, resulting in higher morbidity and mortality rates. In this review, we discuss the different pathologic mechanisms with a focus on epigenetic modifications and their roles in the development and progression of PAH. These modifications include DNA methylation, histone modifications, and microRNA dysregulation. Understanding these epigenetic modifications will improve our understanding of PAH and unveil novel therapeutic targets, thus steering research toward innovative treatment strategies.

## Introduction

**Figure 1:**
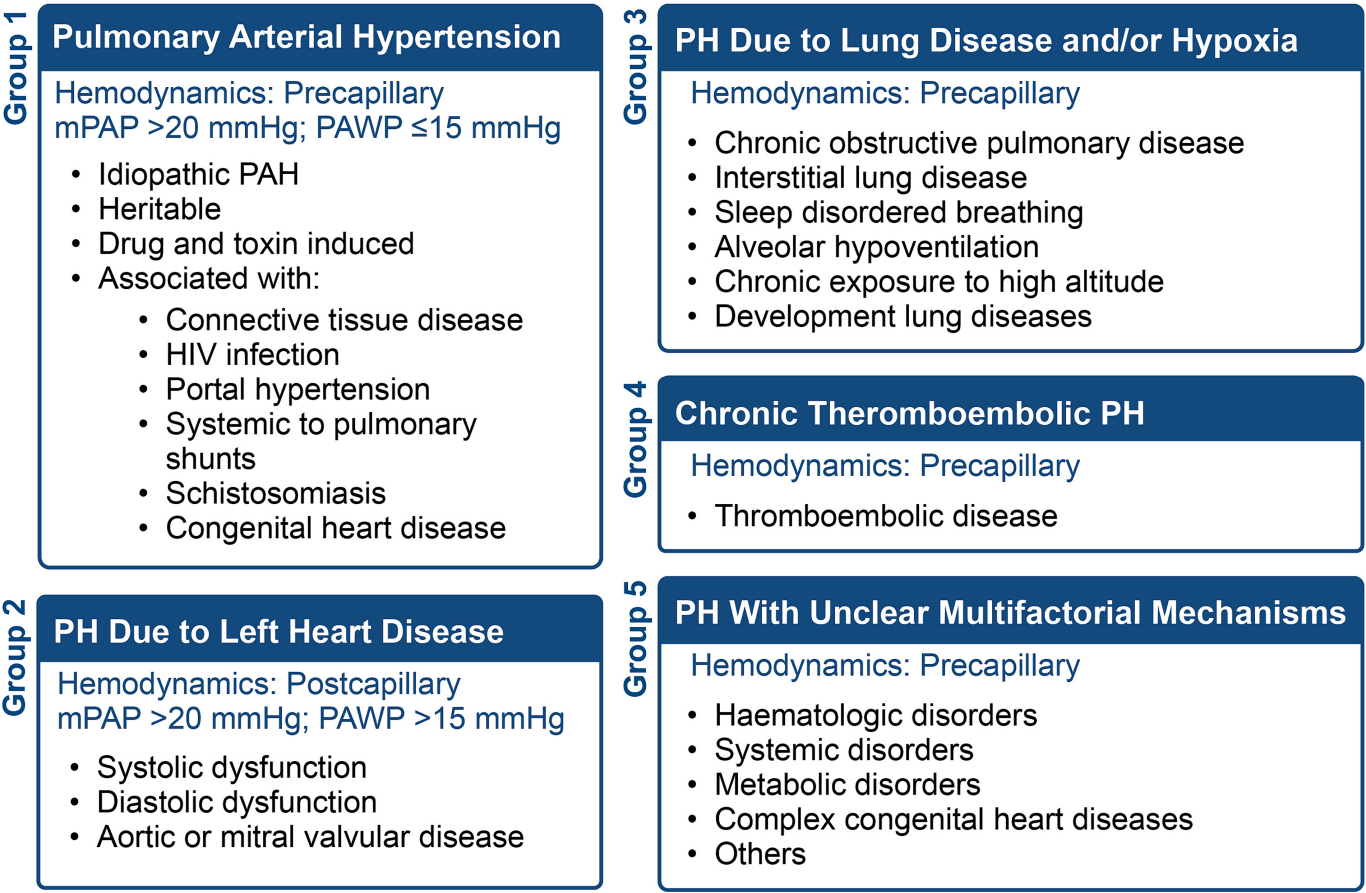
The five WHO classifications of pulmonary hypertension (PH), the hemodynamics, and conditions associated with each group.

Pulmonary vascular disease refers to disorders affecting the lung vasculature, including the pulmonary artery, pulmonary vein, and pulmonary capillaries, leading to elevated pulmonary vascular pressure (i.e., pulmonary hypertension) with subsequent right ventricular failure. This chronic cardiopulmonary disorder is grouped into five classes depending on the etiology and conditions, as summarized in Figure 1. Pulmonary Arterial Hypertension (PAH) represents a subset of PH characterized by precapillary involvement from the hyperproliferation of pulmonary smooth muscle cells (PASMCs) and pulmonary artery endothelial cells (PAECs) (1).

PAH is defined by a mean pulmonary arterial pressure (mPAP) greater than 20 mmHg and a pulmonary capillary wedge pressure (PCWP) equal to or below 15 mmHg (2). It is progressive, debilitating and associated with adverse outcomes and increased mortality (3, 4). PAH primarily affects the pulmonary capillaries, with pulmonary veins largely unaffected, except in cases of pulmonary veno-occlusive disease. According to the Registry to Evaluate Early and Long-term Pulmonary Arterial Hypertension Disease Management in PAH (REVEAL), the largest US-based registry, the estimated seven-year survival rate is 50% (4). A similar trend was also observed in the Pulmonary Hypertension Association Registry (PHAR), where intermediate and high-risk PAH patients had 2- and 3-year mortality rates ranging from 18–20% to 28–55%, respectively (5). Additionally, recent epidemiological studies suggested an increase in PAH, with 5.8 cases per million for PAH incidence and PAH prevalence ranging from 47.6 to 54.7 cases per million (2, 6).

PAH pathophysiology involves a complex multifactorial interplay of genetic alteration, epigenetic modifications and environmental factors. This dynamic interplay results in significant variability in disease expression and progression among patients (7). Given the diverse etiologies and heterogeneity of PAH, understanding the molecular mechanisms underlying the development of PAH becomes essential. Recent evidence emphasizes the importance of epigenetics in the pathogenesis of PAH (7–9). Epigenetic modifications, including DNA methylation, histone modifications, and microRNA deregulation, have been identified as contributors to the pathogenesis of PAH in humans and animals (10). These epigenetic modifications are a critical area of investigation as they may provide more insight into the molecular mechanisms underlying PAH and are potential targets for treatment.

### Pathogenesis of PAH

**Figure 2:**
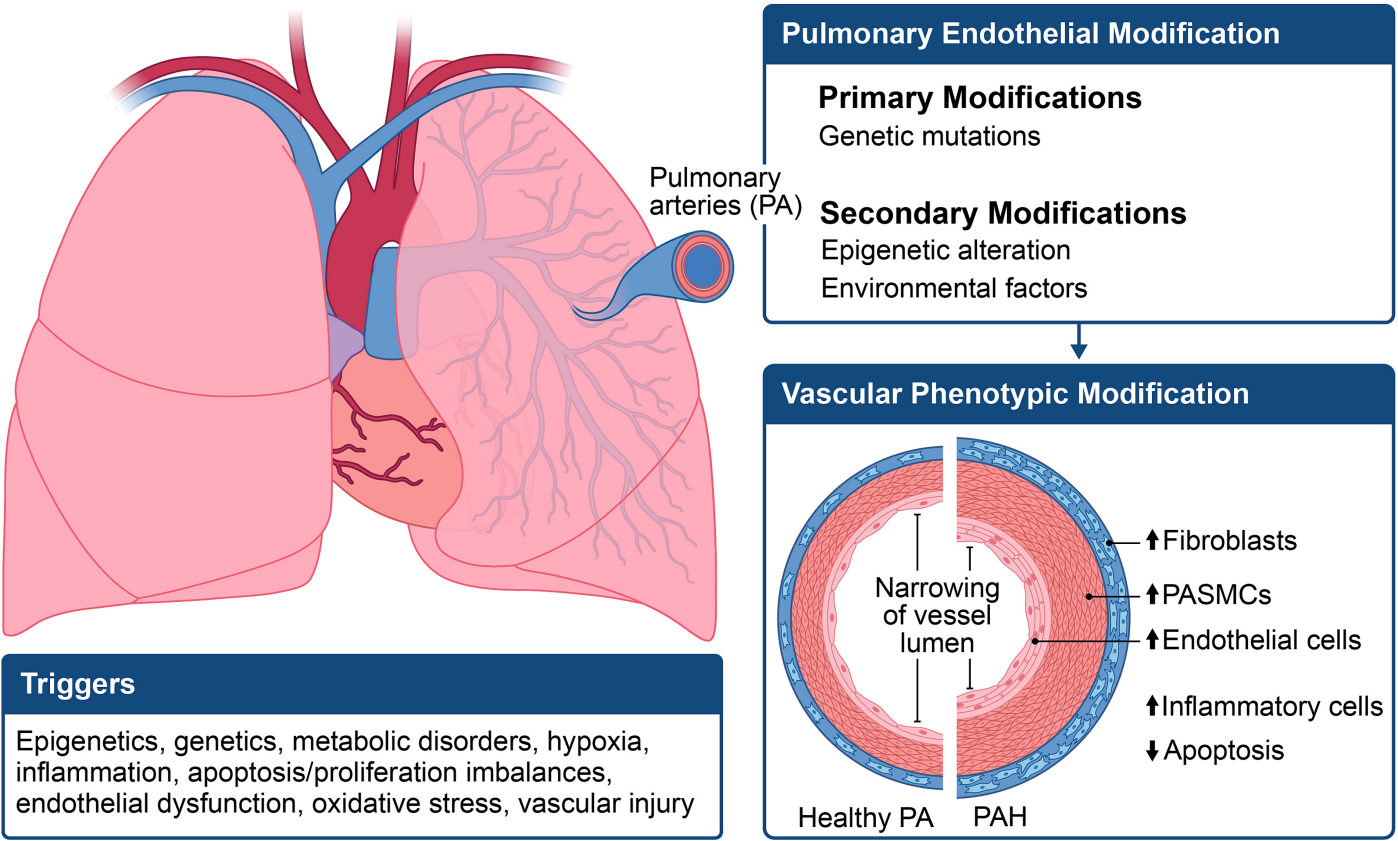
Illustration of the multifactorial mechanisms involved in the pathogenesis of pulmonary arterial hypertension (PAH). The phenotypic expression of PAH results from a combination of genetic, epigenetic, and environmental changes, leading to an increase in pulmonary smooth muscle cell hypertrophy and apoptosis.

The progression of pulmonary vascular disease often begins with the interplay between an initial pathogenic state and one or more triggering stimuli, referred to as the “multiple-hit hypothesis.” Two or more hits could comprise a genetic prediction coupled with an extra genetic factor (such as a mutation or polymorphism), epigenetic modification, comorbidity, stress, or environmental factors (Figure 2). When these factors align, a cascade of effects is set in motion, initiating vascular constriction, cellular proliferation, and a prothrombotic state to varying extents (11, 12). This intricate process culminates in PAH and its associated clinical manifestations. In PAH, pathological vascular remodeling results in distortions in macroscopic and microscopic structures of the pulmonary arterial vasculature. Studies indicate that PAH originates from the abnormal hyperproliferation of pulmonary vascular cells, resulting in neointima formation and narrowing of small distal pulmonary arterioles (13, 14). This luminal narrowing elevates pulmonary vascular resistance, exerting strain on the right ventricle, leading to right heart failure.

Various cell types present in the pulmonary arterial wall contribute to PAH vascular remodeling. Three distinct pathological vascular remodeling processes have been identified as contributors: the muscularization of distal pulmonary arterioles, medial hypertrophy, and neointima formation in proximal muscular arteries, along with the development of plexiform lesions (13, 15, 16).

The muscularization of distal pulmonary arterioles occurs due to the differentiation of progenitor cells into PASMCs, followed by subsequent proliferation (17–19). Progenitor cells involved in this process include stem cells, fibrocytes, or PAECs. Medial hypertrophy and neointima formation also result from differentiating progenitor cells into SMCs, followed by migration and replication in the proximal muscular arteries (20–22). Following muscularization and hypertrophy, there is also abnormal PAEC replication, and the formation of irregular endothelial channels results in plexiform lesions within the narrowed vessel lumen. The PASMCs and PAECs become more resistant to apoptosis, worsening the pathologic vascular remodeling and forming plexiform lesions (23–25).

Another cell type identified in the pathogenesis of PAH is the pulmonary pericyte. Located in perivascular regions, these cells play a crucial role in endothelial cell maturation, immune signaling, and modulation of vascular tone through their impact on cellular remodeling and immune responses (26). The involvement of pericytes in the pathogenesis of PAH has garnered significant research interest. A 2014 study by Ricard et al. revealed an increase in pericytes within the distal pulmonary arteries of human idiopathic PAH (IPAH) endothelial cells compared to *in vitro* control cells. This research underscored the significance of transforming growth factor-β in promoting the differentiation of pericytes into contractile smooth muscle-like cells in distal pulmonary arterioles (27).

Additionally, Yuan et al. demonstrated that PAH pericytes isolated from the lungs of PAH patients exhibited defective association with endothelial tubes *in vitro*, leading to smaller vascular networks as well as narrower endothelial tubes. This defect was attributed to an intrinsic issue in cell motility and polarization, impairing the ability of PAH pericytes to migrate toward vascular tubes and resulting in fewer endothelial-pericyte interactions. The study demonstrated that PAH pericytes are involved in in the loss of small vessels in PAH and suggested that therapeutic approaches targeting the restoring Wnt/PCP activity in these cells could help prevent vessel loss and promote small vessel regeneration in patients with this severe condition (28).

Furthermore, pyruvate dehydrogenase kinase 4 (PDK4) gene and protein expression were found to be significantly elevated in PAH pericytes. This elevation is associated with decreased mitochondrial metabolism, increased glycolysis rates, and hyperproliferation. Notably, lowering PDK4 levels restored mitochondrial function, decreased cell proliferation, and enhanced endothelial-pericyte interactions in PAH models (29).

### Metabolic dysregulation

Metabolic dysregulation plays a central role in PAH, marked by a transition from oxidative phosphorylation to glycolysis, a phenomenon termed the “Warburg effect” (30, 31). This shift leads to notable metabolic alterations, including increased cytoplasmic glycolysis and glutaminolysis, decreased oxidation of fatty acids and disrupted biogenesis of mitochondria (32, 33).

In PAH patients, the increase in cytoplasmic glycolysis, observed in PAEC and PASMCs, compromises the efficiency of ATP generation via the mitochondrial tricarboxylic acid cycle (34–37). Further, PAH is characterized by reduced mitochondrial metabolism, evidenced by reduced mitochondrial abundance, oxygen consumption and mitochondrial DNA in PAEC and PASMC (38–41). This metabolic imbalance leads to compromised mitochondrial respiration elevated reactive oxygen species production, and a reduction in antioxidants like superoxide dismutase 1 and 2, exacerbating the progression of PAH (35, 38, 42).

The “Warburg effect” refers to the inhibition of pyruvate dehydrogenase, leading to decoupled glycolysis and decreased utilization of mitochondrial pyruvate in PAH (41, 43–45). Additionally, various cell types in PAH, such as PAECs, PASMCs, fibroblasts, macrophages, and myocytes of the right ventricle, exhibits reduced mitochondrial glucose oxidation and increased glycolysis (46). The altered metabolism in PAH, affecting glucose homeostasis and vascular remodeling, is associated with the downregulation of the transcription factor PPAR γ, a downstream target of BMPRII (47). Considering the regulatory role of mitochondrial products on transcription factors and epigenetic mechanisms, targeting the epigenetic and metabolism pathway holds promise as a strategy that could be used for PAH therapy (43, 48).

Although less understood, disturbances in fatty acid metabolism are observed in the pulmonary vascular system and right ventricle, resulting in PASMC proliferation, right ventricular steatosis, and lipotoxicity. This is attributed to triglyceride metabolites and reactive oxygen species resulting from elevated fatty acid delivery and synthesis, coupled with diminished oxidative mitochondrial capacity (49, 50).

### Inflammation and immune modulation

Inflammation is a frequently noted phenomenon in PAH (51). Both the innate and adaptive immune systems are impacted by imbalances in inflammatory and immunological responses, which may be influenced by genetic and environmental factors. This anomaly is demonstrated by the perivascular infiltration of dendritic cells, mast cells, T and B lymphocytes, or macrophages situated in close proximity to pulmonary arteries (52).

PAECs and inflammatory cells play crucial roles as both are sources and targets of chemokines and cytokines, contributing to pulmonary vascular remodeling. Proinflammatory cytokines, interleukin 6 and interleukin 1β, directly influence smooth muscle, immune cells, and PAEC migration, proliferation, and differentiation (51, 53–55). In PAH, autoantibodies and local lymph follicles promote inflammation and immunological activation (56, 57). The alterations observed in PAECs and PASMCs in PAH patients create a synergistic effect, intensifying inflammation. Various cell types, including PAECs, PASMCs, myofibroblasts, and fibroblasts, exhibit a significant pro-inflammatory state, evident in the increased expression and release of inflammatory cell-associated cytokines, chemokines, and adhesion molecules, such as E-selectin, vascular cell adhesion molecule 1, and intercellular adhesion molecule 1, are seen. Deactivation of the Forkhead box O1 pathway and elevated levels of interleukins-1 and -6, leukotriene B4, leptin receptors, or tumor necrosis factor all contribute to harmful changes in the pulmonary vasculature via different signaling pathways (58–63).

Similarly, mutations in BMPRII result in heightened levels of proinflammatory cytokine levels, including interleukin 1β and interleukin 6 (51, 53, 64). The BMPR II loss of function results in an increase in fibroblast growth factor 2, enhanced mitogen-activated protein kinase activity, and elevated levels of interleukin 1β, and interleukin 6 and a reduction of vasoprotective peptide apelin in PAECs (53, 54, 65, 66). These cytokines can induce PAECs to produce more fibroblast growth factor 2 (55). They are also essential in coordinating proliferative responses in PASMCs and pulmonary artery fibroblasts because they secrete fibroblast growth factor 2 and interleukin-6 (53, 54, 66). Comparably, as demonstrated by both experimental PH models and human PAH, a disrupted signaling pathway of BMPR 2 can lead to the improper production of growth factors and proinflammatory responses in vascular cells (51, 64, 65, 67, 68).

### Epigenetic mechanisms in PAH

The term “epigenetics” derived from the Greek word “Epi,” meaning on or above, refers to factors beyond the genetic code that modify DNA sequences, inducing phenotypic changes without altering the DNA base pair. Essentially, epigenetic factors influence the transmission and expression of traits not encoded in the genetic material. In humans, mounting evidence highlights the crucial role of epigenetic factors in cellular differentiation, survival, apoptosis, tumor suppression, and genomic imprinting. These factors are induced by environmental exposures and the complex interplay of gene–environment interactions, contributing to various disease states (69, 70). This emphasizes the essential role of epigenetic modifications in the formation of various pathological processes, including tumorigenesis, metabolic disorders, vasculopathy and PAH (71–75).

**Figure 3:**
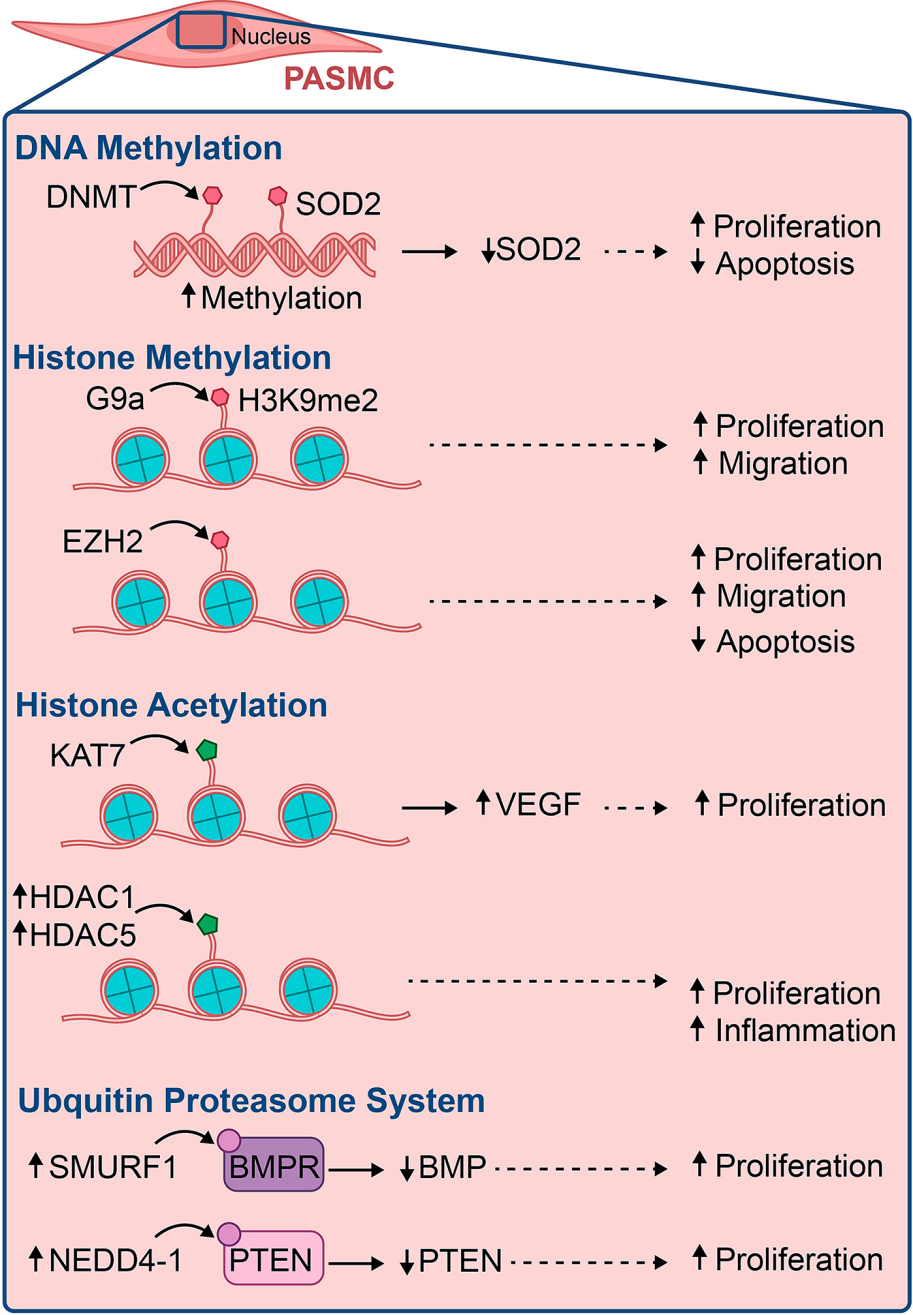
Illustration of some major epigenetic modifications involved in the pathogenesis of pulmonary vascular diseases, their epigenetic marks, and the outcomes that lead to PAH.

PAECs depend on expressing and silencing specific genes to maintain homeostasis. Epigenetic modifications can lead to alterations in these pathways, resulting in dysregulation of PAECs, increased proinflammatory cells, and ultimately, the formation of PAH (76). In PAH, these inheritable changes manifest via three major epigenetic mechanisms: DNA methylation, histone post-translational modification, and non-coding RNA regulation (Figure 3). These modifications are composed of writers, erasers, and readers, directly impact DNA transcription, chromatin packing, mRNA, and protein expression, thereby activating or inhibiting genes (77, 78). We will explore the major epigenetic modifications to understand their roles in the pathophysiology of PAH.

### DNA methylation

DNA methylation involves the covalent addition of a methyl group (-CH3) to adenosine or cytosine residues in CpG islands. The CpG sites are regions in the DNA with specific base pairing, commonly in the promoter regions, that can undergo methylation. Methylated cytosines act as attachment sites for methylated CpG-binding proteins (MeCP) that recognize and bind to methylated CpGs (79). Through the methylation of the CpG islands, proteins involved in gene expression and repression can be directly recruited. CpG methylation also promotes a dense winding of DNA around the histone core, resulting in a structural change (80). Methylation and demethylation are crucial for the regulation of gene expression, cellular differentiation, organ morphogenesis, cell reprogramming, X-chromosome inactivation, RNA splicing, transposon silencing, and DNA repair (79, 80). DNA methylation is mediated by a group of enzymes known as the DNA methyltransferases (DNMT), including DNMT 1, DNMT 2, DNMT3A, DNMT3B and DNMT 3 L (81, 82). In mammalian cells, DNMT1 is the primary methyltransferase implicated in the inheritance of DNA methylation. DNA methylation pattern from parents is copied into newly produced offspring DNA by DNMT1, which interacts with partly methylated DNA during DNA replication (79). DNMT1 exists in an inhibitory state, requiring binding with another protein, E3 ubiquitin-protein ligase (UHRF1). The UHRF1-DNMT complex attaches during the S phase of DNA replication (78). On the other hand, *de novo* DNA methylation requires Dnmt3a and Dnmt3b. They act directly on the CpG sites, adding methyl groups to form new genetic transcription. Methylated CpG islands also interact with methyl-CpG binding proteins (MBDs) via histone deacetylation, playing an essential role in gene silencing (83). Transcriptional repression happens through three mechanisms: (i) nucleosome compaction that obscures transcription factor binding sites, (ii) the recruitment of repressor proteins, and (iii) interaction with histone modification processes (84, 85). Hyper-or hypo-DNA methylation is susceptible to alteration by environmental factors. This phenomenon is noteworthy as it is the “second hit” required to unmask numerous disease conditions by creating a distinct synergistic effect with pathologic genetic mutations (86).

### Ten eleven translocation

The ten eleven translocation (TET) proteins play a crucial role in regulating gene expression by promoting locus-specific reversal of DNA methylation. TET methylcytosine dioxygenases oxidizes 5-methylcytosine (5-mc) to 5-hydroxymethylcytosine (5-hmC), 5-formylcytosine (5-fC), and 5-carboxycytosine (5-caC). These oxidized forms of cytosine enable DNA methylation through both active and passive mechanisms (87–89).

Tet-methylcytosine-dioxygenase-2 (TET2), an essential enzyme involved in DNA demethylation, has recently been linked to pathologic vascular remodeling, inflammation, and clonal hematopoiesis. In IPAH and other PAH-related conditions, TET2 expression was found to be significantly reduced in peripheral blood cells of these patients, making it a potential biomarker for PAH (90).

A study by Potus et al. reported an association between germline mutations in TET2 and PAH (91). Analyzing a PAH biobank of 2,572 cases, the study revealed numerous harmful germline and somatic variants of TET2. The data showed that 0.39% of PAH cases had TET2 abnormalities, with 75% being attributed to germline mutations and 25% to somatic mutations (91). Moreover, 86% of PAH patients exhibited significantly downregulated circulating TET2. These patients were typically older and responded to vasodilatory therapy.

Furthermore, the study revealed that TET2 depletion led to spontaneous development of PAH, as demonstrated by elevated right ventricular systolic pressure, increased total pulmonary resistance, reduced pulmonary artery acceleration time, adverse remodeling of the pulmonary vasculature, and inflammation (91). Further analysis of Tet2 hematopoietic conditional knockout (KO) mice and heterozygous Tet2+/− mice revealed that even partial Tet2 deletion could induce PAH, indicating a gene dose-effect response (91).

D’Addario et al. (92), examined differences in leukocyte expression of DNMT and TET and the severity of PAH among various ethnic groups. Their findings revealed that PAH patients had higher expression levels of DNMTs (3a and 3b) and TETs (2 and 3) compared to healthy controls, with notable ethnic variations (92). Specifically, the study found that TET2/TET3 expression levels were higher in Hispanic and African American patients diagnosed with scleroderma-associated and IPAH compared to Caucasians, whereas DNMT1 expression was downregulated in these patients (92). Additionally, the study found higher levels of inflammatory cytokines IL6 and CCL5 in Caucasian PAH patients compared to Hispanic/African American patients. The altered DNA methylation and reduced TET expression may be associated with elevated inflammatory cytokines and hematological disorders or malignancies.

#### DNA methylation in PAH

In PAH, DNA methylation in PAEC influences the regulation and function of endothelium-specific expression, mediated by VEGF signaling pathways (93–95). The eNOS enzyme is crucial for producing nitric oxide within the vascular endothelium cells, which is essential for facilitating vasodilation and preserving endothelial homeostasis (95). Methylation of the DNA at the eNOS proximal promoter can impair its functional activity, affecting vascular tone and angiogenesis (93).

Research showed that fawn-hooded rats exhibited heightened levels of DNMT1 and DNMT3B, along with hypermethylation of the CpG island of the superoxide dismutase (SOD)2 promoter, in both lung tissue and isolated PASMCs (8). This silencing of SOD2 transcription activates hypoxia-inducible factor (HIF)1, leading to hyperproliferation, apoptosis resistance, and the subsequent spontaneous development of PAH (8). Notably, the DNA methyltransferase inhibitor 5-aza-2′-deoxycytidine selectively reversed DNA methylation, restoring SOD2 expression and the ratio of proliferation to apoptosis in the lungs of fawn-hooded rats (8).

Yang et al. found that long-term high-altitude hypoxia in fetal lambs led to reduced DNA methylation levels and, subsequently, a loss of the cyclin-dependent kinase inhibitor p21 in the pulmonary vasculature. These changes are likely responsible for the pulmonary arterial remodeling.

and PH was observed in newborn lambs (96). Furthermore, DNA methylation is also implicated in activating or inhibiting inflammatory pathways associated with PAH (97–99).

### Histone post-translational modification

Since the first histone methyltransferase was discovered in the 2000s, histone post-translational modification has been a great field of research interest in vascular biology (100–103). The nucleosome core particle of chromatin is made up of a histone protein octamer with 146 base pairs of DNA organized into a superhelix around it. The nucleosome is formed by two sets of histone octamers composed of H2A, H2B, H3, and H4 proteins, and is further condensed by linker DNA (104, 105). Histones are post-translationally modified in the histone tail, which consists of 10–30 amino acids at the N-terminal domain. Post-translational modification modulates chromatin folding and regulatory protein binding within the nucleosome to produce histone variants that modulate the transcription and expression of genes (106). Various histone post-translational modifications involve histone embryonic vascular development, remodeling, and various vascular disease pathologies (107).

The primary histone modifications of note include methylation and acetylation, processes where small organic molecules are added to the histone tail through chemical mechanisms. Other such modifications include phosphorylation, deamination, and palmitoylation. The second group of histone modifications involves the addition of larger organic molecules to the histone tail, including ubiquitylation, SUMOylation, biotinylation, glycosylation, and ADP-ribosylation (108, 109).

### Histone methylation

Histone methylation is a reversible post-translational modification involving two enzymes: histone methyltransferase (HMTs) as the writer and histone demethylases (HDMTs) as the eraser. Histone methylation involves the addition of -CH3 groups to the amino acids at the histone tail. This process tightly modulates gene expression depending on how many methyl groups and/or which amino acid is attached. The HMTs are classified into two groups: protein lysine methyltransferases (PKMTs) and protein arginine methyltransferases (PRMTs) (110, 111). Multiple methyl groups may be added by this post-translational alteration, leading to monomethylation (me1), dimethylation (me2), and trimethylation (me3). The most common epigenetic modification observed in histone methylation is gene silencing via polycomb group proteins (PcG) (112). These proteins regulate cellular differentiation by inhibiting inappropriate cellular activation and proliferation. In mammals, PcG proteins organize into two main complexes: polycomb-repressive complex 1 (PRC1) and 2 (PRC2). The PRC1 is made up of the BMI1, RING1A/B, CBX, and PHC subunits (113). PRC1 functions by binding to nucleosomes, resulting in chromatin compaction (114). Conversely, PRC2 is composed of EZH2 or EZH1, EED, SUZ12, and RbAp46 (113–115). EZH2 is extensively researched as the catalytic subunit of PRC2, housing the SET domain crucial for tri-methylation of lysine 27 on histone H3 (H3K27me3), resulting in transcriptional inhibition (113). PcG-dependent gene silencing involves a synergist effect between PRC1 and PRC2 complexes. Once the PRC2 complex forms H3K27me3, it recruits PRC1 by binding the chromodomain of the PHC subunits (116). Chromodomains found within the HP1 proteins recognize di-and tri-methylated histones (H3K9), which results in the recruitment of heterochromatin protein 1 (HP1) to sites of repressed chromatin. Similarly, CBX proteins, containing a sequence structurally similar to the N-terminal of the chromodomain, are recruited to di-or tri-methylated H3K27, forming a multi-protein complex that regulates genes associated with development and differentiation. The CDY1 gene encodes a protein that includes both a chromodomain and a histone acetyltransferase catalytic domain that binds to H3K9me resulting in gene repression (112). Another chromodomain, CHD1, has been shown to recognize H3K4me, resulting in gene transcription. Independent of H3K27me3, EZH2, and other PRC2 subunits have been identified in the cytoplasm, where they modulate actin polymerization and cell proliferation of T lymphocytes and fibroblasts (117).

Bromodomains and chromodomains are protein domains capable of identifying and attaching to modified amino acids on histone tails. The bromodomain and extra-terminal (BET) domain family are known as epigenetic readers and have been linked to numerous diseases, as evidenced by recent studies (118–122). This family includes BRD2, BRD3, BRD4, and testis-specific bromodomain proteins, all interacting with other proteins and acetylated histone tails through their tandem bromodomains 1 and 2. BET proteins regulate the transcription of genes involved in various biological processes such as cell division, apoptosis, and inflammation (123). Among these, BRD4 is extensively studied in current research on PAH.

BRD4 modifies the chromatin structure and facilitates the transcriptional activation of target genes by acting as a scaffold for transcription factors at promoters and super-enhancers (124). It stimulates proinflammatory endothelium genes such as IL-6 (interleukin-6), tumor necrosis factor-α, and monocyte chemoattractant protein-1 and is also linked to vascular remodeling (123, 125). This activation is further stimulated through a feedback loop (126). These proinflammatory cytokines have been shown to damage DNA and found to be increased in PAH (127, 128).

Furthermore, Meloche et al. found that, in comparison to controls, BRD4 was upregulated in the lungs, distal pulmonary arteries, and PASMCs of patients with PAH, indicating that BRD4 is dependent on miR-204 (124). Three key oncogenes overexpressed in PAH were reduced in expression by pharmacological suppression of BRD4 using JQ1 or siBRD4: nuclear factor of activated T cells, B-cell lymphoma 2, and survivin. Inhibiting this oncogenic signature resulted in a BRD4-dependent reduction in PAH-PASMC proliferation accompanied by an elevation in apoptosis, suggesting that BRD4 overexpression is pathologically associated with PAH and may be a target for therapy (124).

Therefore, through regulating gene expression, BRD4 is an essential regulator of the equilibrium between proliferation and apoptosis and is involved in post-DNA damage events, making it a potential target for the development of PAH therapy (129).

#### Histone methylation in PAH

A study by Yang et al. showed that inhibition of histone lysine methyltransferase G9a (HMT G9a), an enzyme necessary to generate H3K9me2/histoneH3 dimethylation at position lysine-9, by HMT G9a inhibitor BIX-01294 resulted in PDGF-induced proliferation, migration, and contractility in ovine pulmonary arterial smooth muscle cells (117). Similarly, another study showed that EZH2 overexpression was correlated with an increase in right ventricular pressure and hypertrophy in a hypoxia-induced mouse model (130). These studies highlight the possible epigenetic mechanisms of the development of PAH through dysregulation of histone methylation. This creates an area for advancement in the treatment of PAH by targeting protein methyltransferases or demethylases.

### Histone acetylation

An additional post-translational modification of histones is the histone acetylation. It involves adding negatively charged cofactor acetyl groups to activate gene expression (131). Histone acetylation is catalyzed by histone acetyltransferase (HAT) enzymes, which transfer the acetyl groups to the positively charged lysine residues of the histone tail. The neutralization of the lysine residue weakens charge-dependent interactions between the histone and DNA, allowing for transcription. There are two types of HATs: Type A located in the nucleus and Type B located in the cytoplasm. They work together to acetylate histones for transcriptional activation and replication, respectively, before the chromatin is assembled (132). The most active HATs in mammals include HIV Tat interactive 60-kDa protein (Tip60), p300, CREB binding protein associated factor (PCAF), cAMP response-element binding protein (CREB), and CREB binding protein (CBP) (133). HATs like histone acetyltransferase 7 (KAT7) have been identified to regulate endothelial function in zebrafish embryos by initiating the transcription of vascular endothelial growth factors (VEGFs) (134).

Conversely, histone deacetylation uses histone deacetylases, or HDACs, to control the amount of histone acetylation. The four families of histone deacetylases (HDACs) are responsible for removing acetyl groups from histone proteins. (i) The nucleus contains class I HDACs (HDACs 1, 2, 3, and 8), (ii) The cytoplasm and nucleus express class II HDACs (HDACs 4, 5, 6, 7, 9, and 10), (iii) The nucleus contains class III HDACs (Sirtuins 1–7), and (iv) both the cytoplasm and the nucleus express class IV HDACs (HDAC 11) (135). The sirtuins (SIRT) regulate a number of processes, such as aging, stress damage, metabolism, transcription, and cell division (136). Overall, HDACs silence DNA transcription by tightening chromatin wrapping.

#### Histone acetylation in PAH

Abnormal histone acetylation and deacetylation, crucial processes in the regulation of gene expression, have been identified in pulmonary hypertension. Zhao et al. demonstrated that higher levels of HDAC1 and HDAC5 were observed in the lungs of IPAH patients (7). Similarly, isolated fibroblasts showed an increased expression of class I HDACs (HDAC1, HDAC2, and HDAC3) from the distal pulmonary arteries of chronically hypoxic hypertensive calves (137). In line with these, another study shows increased H3 and H4 acetylation at the proximal promoter region of eNOS, resulting in elevated levels of eNOS in PAECs isolated from persistent PH of the newborn (PPHN) rats, highlighting the significant role of epigenetic modification in the pathogenesis of PPHN (138).

Hypoxia, a well-recognized trigger for lung vascular remodeling as well as endothelial dysfunction, is crucial for the development of PAH (139–144). The hypoxia signaling pathway operates through hypoxia-inducible factors (HIFs), which stabilize in cells under low oxygen conditions and subsequently activate hypoxia-responsive elements (HRE) in the promoters of target genes (145). Humans express three HIF isoforms: HIF-1α, HIF-2α, and HIF-3α, each exhibiting distinct cell-specific functions (146). Under normoxic conditions, HIF-1α and HIF-2α are quickly degraded, however, low oxygen levels prevent their degradation, which plays a role in the onset of PAH (147, 148). HIF-1α plays a pivotal role in the body’s response to low oxygen levels by stimulating the transcription of genes involved in angiogenesis, vasculogenesis, apoptosis, and energy metabolism (149–151). Important targets of HIF-1α include vascular endothelial growth factors (VEGFs), which interact with and activate receptors that promote angiogenesis, thereby increasing vascular permeability (151, 152). Conversely, HIF-2α uniquely promotes endothelial-to-mesenchymal transition (EnMT), aiding in pulmonary vascular remodeling (148). HIF-1α expression increases rapidly with hypoxia, while HIF-2α accumulates more gradually (147).

HIF transcription factors impact inflammation and epigenetics by activating NF-κB signaling through prolyl hydroxylases that regulate HIF activity and degradation (146). Hypoxia and HIFs activate significant functional shifts in ECs during PH, including metabolic reprogramming, dysfunction of endothelial colony-forming cells, compromised angiogenesis, and alterations in estrogen metabolism facilitated by HIF-1α (35, 65, 153, 154). Recent findings highlight HIF-2α as a significant driver of lung vascular remodeling and PH, promoting EnMT, where ECs transform into myofibroblast/SMC-like cells (155–157). EnMT promotes remodeling of the lung vascular and plays a role in the development of dysfunctional endothelial phenotypes in PAH (158, 159). Importantly, the regulation of EnMT involves epigenetic modifications of numerous target genes. Hypoxia and HIFs trigger a range of epigenetic alterations, including histone acetylation and methylation (7, 160).

Another study by Paulin et al. demonstrated that a decrease in SIRT3, a mitochondrial deacetylase, was linked to the suppression of mitochondrial function, prevention of apoptosis, and activation of several transcription factors related to pulmonary hypertension. HIF1α, signal transducer and activator of transcription 3 (STAT3), and nuclear factor of activated T-cells (NFAT)c2 have been identified (48, 106).

Given these findings, several compounds targeting these histone acetylation and deacetylation mechanisms have been explored as possible therapeutic options. In 2010, Cho et al. evaluated the therapeutic effects of valproic acid (VPA), a commonly used mood stabilizer with HDAC class 1 inhibitor activity, on rat models with monocrotaline (MCT)-induced PAH and right ventricular hypertrophy (RVH). The authors found that VPA administration effectively inhibited MCT-induced RVH and improved RV systolic function (161). Similarly, in 2012, Zhao et al. mitigated the development and pulmonary vascular remodeling of hypoxia-induced pulmonary hypertension using both VPA and suberoylanilide hydroxamic acid (vorinostat), an inhibitor of class I, II, and IV HDAC (7, 162).

Conversely, a study by Boggard et al. using a different broad-spectrum HDAC inhibitor, trichostatin A (TSA), did not prevent RVH but resulted in increased myocardial cell death, RV dysfunction, and fibrosis in rat models (163). This discrepancy in findings about the effects of broad-spectrum HDAC inhibitors is likely due to side effects from other pharmacological activities. This led to further evaluation of selective HDAC inhibitors as a better therapeutic option for PAH treatment to mitigate side effects and limit the target of additional genes. So far, Kim et al. showed that selective HDAC class IIa inhibition augmented MEF2 activity, a factor that regulated the expression of various transcriptional targets, including microRNAs 424 and 503, connexins 37 and 40, and Krűppel Like Factors 2 and 4, involved in pulmonary vascular homeostasis, and protected against RV dilatation (9). Relatedly, Cavasin et al. demonstrated that a benzamide HDAC inhibitor, MGCD0103, a selective inhibitor of class I HDACs 1, 2, and 3, reduced pulmonary arterial pressure and vascular remodeling more dramatically than tadalafil (164). Abnormal histone acetylation and deacetylation have been identified in PH. Zhao et al. illustrated that higher levels of HDAC1 and HDAC5 were observed in the lungs of IPAH patients (7). Similarly, isolated fibroblasts showed an elevated expression of class I HDACs (HDAC1, HDAC2, and HDAC3) from distal pulmonary arteries of chronically hypoxic hypertensive calves (137).

### Ubiquitination and proteasome activity

The Ubiquitin Proteasome System (UPS) is essential for several cellular processes, such as protein transport and breakdown, transcription control, and DNA repair. All tissues of eukaryotic organisms have the 76-amino acid protein known as ubiquitin (165). The ubiquitination process involves adding a C-terminal group of ubiquitin to a lysine residue of the substrate, for example, the histone tail, resulting in epigenetic modification. Ubiquitination is a multistep ATP-dependent process mediated by three essential enzymes, E1, E2, and E3 enzymes, facilitating the attachment of ubiquitin monomers or chains to proteins (166). Similarly, the proteasome system, which is made up of the 20S core proteasome (CP) and the 19S regulatory particle (RP), is part of this process. The RP governs substrate protein binding and ubiquitination into the CP through its primary structure containing ATPase and its cap structure without ATPase. The process of ubiquitination and de-ubiquitination is reversible and highly controlled, as dysregulation in this complex has been linked to numerous medical conditions (167).

#### Ubiquitin proteasome system in PAH

Under stress conditions, like oxidative stress, these proteasomes can be mobilized to increase ubiquitination rates to remove damaged proteins (168). Numerous studies highlight the close association between the UPS and the initiation and progression of PAH. While the mechanism is not fully understood, research indicates that several E3 ubiquitin ligases, including SMURF1, NEDD4, and SIAH2, are implicated in PAH development in patients or experimental models, particularly related to altered protein ubiquitination. In HPAH with BMPRII gene mutations, SMAD ubiquitination regulator 1 (SMURF1), a member of the HECT family of E3 ubiquitin ligases, targets BMPR, resulting in degradation and downregulation of the downstream cascade (169). This reduction in BMP levels induces endothelial cell apoptosis and SMC proliferation (170, 171). Additionally, pulmonary vascular cells from PAH patients and animal models have elevated levels of SMURF1, suggesting a role of SMURF1 in the pathogenesis of PAH (172).

Furthermore, NEDD4-1, a PTEN E3 ligase involved in cell migration and tumorigenesis, was found to have increased levels in MCT-induced PAH rat models (173, 174). These increased levels activate the PI3K/AKT signaling pathway, which is involved in the proliferation of human smooth muscle cells and vascular remodeling (173). Research on the UPS has been crucial in developing potential PAH treatment targets. Bortezomib (BTZ), an FDA-approved proteasome inhibitor, demonstrated efficacy in reversing pulmonary vascular remodeling in PAH rats (175). Unfortunately, its use is limited as it causes global apoptosis in both right and left ventricles in the PAH rat model, limiting its clinical use. Carfilzomib (CFZ), another proteasome inhibitor, was effective in treating PAH in human pulmonary vascular cells when used in combination with vasodilators. It effectively reversed pulmonary vascular remodeling and induced apoptosis (176). However, the reports are mixed, as other studies suggest CFZ may induce PAH in cancer patients. Even though none of these are being used in clinical settings, they do point to a possible target for PAH treatment.

### Non-coding RNAs

Another layer of epigenetic modifications is through non-coding RNAs (ncRNAs). They are typically classified into two groups based on size: small ncRNAs (<200 nucleotides) and long non-coding RNAs (lncRNAs) (>200 nucleotides). The small ncRNAs are made up of microRNAs (miRNAs), piwi-interacting RNAs (piRNAs), and small-interfering RNAs (siRNAs). Conversely, LncRNAs, consist of small nucleolar RNAs (snRNAs) and natural antisense transcripts. LncRNAs are gene transcripts that directly bind to non-coding regions of mRNAs to modify the expression of those genes (177).

### MicroRNAs

miRNAs are small non-coding genetic transcripts with ~25 base sequence that regulate gene expression. Numerous miRNA genes have been found in eukaryotic organisms, making them one of the largest gene families (178). The regulatory network of miRNA activity is intricate, given that an individual miRNA can bind to and control the expression of numerous genes. Conversely, multiple miRNAs can bind to and regulate a single mRNA target (179). The RNA polymerase III (pol III) transcribes pri-miRNA, a long primary transcript from the nucleus to pre-miRNA. The pre-mRNA maturation occurs through a sequential process. The pre-mRNA, which is several hundred base pairs long, is processed by the RNAse III endonuclease Drosha into a smaller fragment of 60–70 nucleotides, which is then transported to the cytoplasm (180, 181). The pre-miRNA is then processed by another RNA endonuclease, Dicer, to produce a mature double-stranded miRNA. The mature miRNA is separated and incorporated into a ribonucleoprotein complex known as the RNA-induced silencing complex (RISC) (182). The incorporated miRNA plays a crucial regulatory role in various cellular processes, such as hematopoietic cell differentiation, cell proliferation, apoptosis, and organ development (179, 180). miRNAs have been shown to regulate critical signaling pathways involved in the proliferation and migration of both PASMCs and PAECs necessary in the pathogenesis of PAH.

#### MicroRNAs in PAH

Epigenetic modifications of miRNAs have been implicated in several key signaling pathways associated with PAH. These pathways include BMPRII/TGβ, the H1F-signaling axis, the PPAR-γ signaling axis, IL-6/STAT3 and others. The dysregulation of the BMPRII/ALK1 signaling pathway is implicated in the development of HPAH and IPAH. Numerous signaling proteins in the BMP/SMAD pathway are influenced by different miRNAs. For example, miR-140-5p targets the SMURF1 protein, activating of the BMP/SMAD signaling and promoting migration and proliferation of PASMCs *in vitro* (172). Similarly, miR-23a promotes hypoxia in PASMCs by suppressing the expression of BMPRII, which causes PASMC hyperproliferation and formation (172, 183).

miR-98-5p targets ALK1, a transmembrane serine/threonine receptor kinase that suppresses BMPRII. In hypoxia, ALK1 suppression by miR-98-5p led to the downregulation of BMPR2 signaling in PASMC (184). miR-17-5p and miR-20a also repress BMPRII expression, influencing its regulation in the IL-6/STAT3 signaling axis, an important inflammatory pathway implicated in PAH pathology (184, 185).

Several miRNAs target tyrosine kinase receptors are the target of, which activate downstream cascades and affect cell survival and proliferation. MicroRNA analysis revealed microRNA-193-3p and 3p and miRNA-328 were significantly downregulated in the lung tissue of patients and rodents with PH, leading to posttranslational inhibition of Type 1 insulin-like growth factor receptor (IGF1R) (186, 187).

Additionally, hypoxia is a prominent inducer of proliferative vasculopathy in animal models of PH (188, 189). Cellular oxygen sensing is complex and tissue-specific, with hypoxia-inducible transcription factors, HIF-1α and HIF-2α, playing crucial roles in pulmonary vascular cells. Chronic hypoxia stabilizes HIF-1α, leading to upregulation of various miRNAs including miR-1, miR-9, miR-17/92, miR-21, miR-27a, miR-27b, miR-138, miR-190, miR-199-5p, miR-210, miR-322, miR-361-5p and miR-23a (183, 185, 190–200). These miRNAs influence multiple signaling pathways, including transmembrane ion channel function and BMP/SMAD signaling. HIF-1α also regulates various metabolic genes. Hence, the upregulation of HIF-1α induces metabolic shifts favoring aerobic glycolysis in pulmonary vascular cells (201). The significance of endothelial HIF-2α subunits has also been demonstrated in animal PH models. Similarly, miRNA upregulation of Hif2a increasing the expression of arginases, including endothelial Arg 1, resulting in the dysregulation of the eNOS pathway (202, 203).

The PPAR-γ signaling axis is another miRNA target for epigenetic modification. Many miRNAs target PPARγ, and vice versa. Hypoxia stimulates the downregulation of PPARγ *in vitro*. Conversely, research indicates that exposure to hypoxia leads to the elevation of miR-27a, promoting increased proliferation of PAEC, increased expression of endothelin-1 (ET-1), and a reduction in PPARγ expression. These outcomes were counteracted through the inhibition of miR-27a (193). Similarly, hypoxia upregulates the miR-130/301 family, targets PPARγ resulting in the modulation of STAT3-miR-204 axis and an increase in SMC proliferation (204).

Vascular remodeling and PASMC enlargement are further characteristics of PAH. Voltage-dependent potassium channels: KCNA5 is also a target of miRNAs. Increased expression of miR-190 represses KCNQ5, resulting in PASMC cell hypertrophy and reducing its activity and expression (190, 196).

miRNA-138, a significant microRNA, plays a crucial role in the proliferation, differentiation, and apoptosis of PASMCs, signifying its contribution to the advancement of PAH. miRNA-138 specifically targets the potassium channel subfamily K member 3 (TASK-1) and is expressed in PASMCs, resulting in its downregulation under hypoxic conditions in PAH.

### Long non-coding RNAs

Like miRNA, lncRNAs are processed by the RNA processing apparatus after being transcribed by RNA polymerase II or III. They may be multiexonic, 5′-capped, or polyadenylated. LncRNAs are in the nucleus or cytoplasm, where they regulate transcriptional and posttranscriptional gene expression (205). In the cytoplasm, lncRNAs compete with mRNAs for miRNA binding and hence reduce the mRNA-destabilizing potential of miRNAs (206). On the other hand, inside the nucleus, lncRNAs regulate gene expression at the epigenetic level by modulating transcription (205).

#### Long non-coding RNAs in PAH

Similar to miRNAs, hypoxic stress upregulates or downregulates the expression of lncRNAs. Among the lncRNAs expressed in PAECs in PAH, metastasis-associated lung adenocarcinoma transcript 1 (MALAT1) plays a crucial role by modulating the inflammatory cytokines IL-6 and TNF-a (207). Studies demonstrate that hypoxia increases MALAT1, which controls the phenotypic transition and promotes PAEC proliferation in PAH (208).

Hypoxia also stimulates the expression of lncRNA H19. It enhances the proliferation of pulmonary artery smooth muscle cells via AT1R by sequestering let-7b in monocrotaline-induced PH rat models (209). Similarly, hypoxia triggers the expression of urothelial carcinoma-associated protein 1 (UCA1), which acts to bind ING5 away from hnRNP I. This leads to increased proliferation of PASMCs and resistance to apoptosis (210).

Hypoxia upregulates the expression of PAXIP1-AS1, which interferes with the focal adhesion, affecting multiple IPAH-specific transcriptional genes (211).

Finally, hypoxia triggers the upregulation of the long non-coding RNA (lncRNA) HOXA-AS3, facilitating the proliferation and migration of PASMC. HOXA-AS3 stimulates the expression of HoxA3, resulting in heightened levels of cyclins A, E, and D, as well as PDE5A, achieved by the downregulation of miR-675-3p (212).

Hypoxia causes the downregulation of several additional lncRNAs. For example, MEG3 is markedly downregulated in PAH patients’ lungs and pulmonary arteries. Decreased expression of MEG3 leads to increased cell cycle proliferation from the G0/G1 phase to the G2/M + S phase and PASMC proliferation. Notably, this implicates p53 in MEG3-induced smooth muscle cell proliferation (213, 214). Under hypoxic conditions, MEG3 expression is suppressed due to the activation of Cyclin A, PCNA, and Cyclin E, prompting the transition of PASMCs from the G0/G1 phase to the G2/M + S phase through multiple pathways (214). The inhibition of MEG3 leads to the increased expression of miR-21 and the inhibition of PTEN expression (215, 216).

Similarly, lncRNA GAS5 is downregulated in hypoxia-induced PH PASMCs *in vitro*. The study showed that miR-23b-3p directly interacted with Gas5 by targeting its miRNA-binding site, modulating KCNK3 expression. The Gas5/miR-23b-3p/KCNK3 axis provides further insight into hypoxia-induced PASMC proliferation and migration providing potential avenues for future PH treatment (217, 218).

### Estrogen signaling and epigenetics

The impact of sex disparities on PAH is substantial, leading to influencing its prevalence, pathogenesis and treatment responses. Strikingly, there is epidemiological variation, with a 4:1 ratio of females to males, generating curiosity in how estrogen and its metabolites function in the pathogenesis of PAH. Therefore, exploring the therapeutic implications of estrogen inhibitors has gained attention (219–222).

Through estrogen receptors, estrogen signaling decreases BMPRII expression and increases PASMC proliferation in experimental models (223). Although females exhibit a higher incidence of PAH, paradoxically, a worse prognosis is associated with males. This discrepancy is likely attributed to the advantageous effects of estrogen on the right ventricle, commonly known as the “estrogen paradox,” thus making it more challenging to use drugs that target estrogen signaling therapeutically (224–226).

Estrogen is one of the sex hormones that directly affects the mitochondria’s functional dynamics in tissues related to PAH development. Estrogen-receptor-equipped mitochondria contribute to PAH by promoting nuclear respiratory factor-1 (NRF-1) transcription, which increases the amount of mitochondrial transcriptional factors (TFAM) (227). These mitochondrial processes are essential to the pathogenic mechanisms of PAH, since they affect vascular function and cause pulmonary vasoconstriction (228, 229). The role of mitochondria in vascular cells is, at least partially, responsible for the proliferative and antiapoptotic features seen in PAH (229).

Moreover, mitochondria possess the capacity to control the dispersion of reactive oxygen species originating from the mitochondria, impacting their distribution to the cytoplasm and plasma membrane. This, in turn, stimulates redox-sensitive targets like Hif-1 or the NFAT signaling pathway, ultimately resulting in the contraction of pulmonary arterial smooth muscle cells (229).

The transmission of mitochondrial DNA to offspring suggests that female mitochondria are responsive to the changes in their environment that are subsequently inherited by the next generation (230).

Emerging evidence indicates that estrogen may influence the epigenetic modifications involved in the pathophysiology of PAH through mechanisms involving miRNA and lncRNA. Research indicates that estradiol engages in various genomic and non-genomic processes via estrogen receptor alpha (ERα) and beta (ERβ) (231). ERα binds to regulatory regions of target genes, recruiting co-regulatory proteins, and inducing chromatin modifications that can enhance or suppress gene transcription. This enables estrogen to regulate the expression of various miRNAs implicated in PAH (232). In a study by Wallace et al., the researchers examined whether miRNAs expressed in PASMCs are affected by estrogens and play a role in PASMC proliferation. They found that estradiol downregulates the expression of miR-96, which regulates the 5-hydroxytryptamine 1B (5-HT1B) receptor, a mediator of pulmonary artery SMCs. Decreased miR-96 expression was observed in PASMCs of female BMPR II mutant mice and female PAH patients, leading to increased 5-HT1B expression and 5-HT-driven proliferation (233).

Additionally, a study by Mair et al. explored the role of sex, estrogen, and BMPR-II protein mutation in PAH. The study demonstrated that estrogen-driven suppression of mRNA, BMPR-II, and Smad1 signaling in PASMCs of non-PAH females contributed to a pro-proliferative phenotype in hPASMCs, potentially predisposing women to PAH. Furthermore, small interfering RNA (siRNAs) silencing of Smad1 revealed proliferative responses to BMP4 in male PASMCs, with estrogen decreasing messenger RNA and protein expression of Id genes, which are involved in BMPR-II signaling (234). While the role of estrogen in PAH pathophysiology is well-documented, the extent to which estrogen influences PAH and the right ventricle remains unclear.

#### DNA damage and repair in PAH epigenetic

Recent studies have emphasized the crucial role of DNA damage and repair mechanisms in the epigenetic regulation of PAH. These processes can trigger epigenetic changes linked to DNA repair and DNA damage response (DDR) activation. DNA damage, including single-stranded DNA breaks (SSBs), modified bases, double-stranded DNA breaks (DSBs), and inter-and intra-strand crosslinks, can influence the epigenetics of PAH (235). Continuous exposure to cellular metabolites and environmental agents can compromise the integrity of the DNA structure (236). For instance, the DNA damage response can alter histone modifications and DNA methylation patterns, impacting gene expression profiles involved in vascular remodeling and inflammation. Epigenetic modifications, such as hypermethylation of specific genes, have been implicated in the pathogenesis of PAH, contributing to the proliferative and anti-apoptotic phenotype of PASMCs (237). Furthermore, in most plexiform lesions microdissected from idiopathic PAH lung tissues, PAECs were monoclonal, indicating each lesion originated from the proliferation of a single EC (238, 239). Similar findings were observed in patients with PH associated with appetite suppressant, whereas lesions in patients with congenital heart disease-associated PAH (CHD-PAH) or connective tissue disease-associated PAH (CTD-PAH) exhibited polyclonality (238, 239). Subsequently, a study confirmed microsatellite instability within the PAECs of plexiform lesions in PAH patients, supporting this hypothesis (240). Patients with PAH showed microsatellite instabilities in genes such as transforming growth factor-β receptor II (TGFBR2), and BCL-2 associated X, apoptosis regulator (BAX) genes, which play essential roles in controlling cell proliferation and apoptosis.

A study by Perez et al., using whole-exome sequencing, identified genes implicated in IPAH and revealed that topoisomerase-II binding protein 1 (TOPBP1), which is crucial for DNA damage response and replication, was downregulated in pulmonary microvascular endothelial cells (PMVECs) from IPAH patients, resulting in increased DNA damage and apoptosis (241). The most direct link between environmental agents and the development of PAH comes from studies on pulmonary veno-occlusive disease (PVOD), a rare and severe form of PH. Perros et al. demonstrated a significantly higher annual incidence of PVOD in cancer patients treated with mitomycin-C (MMC) compared to the general population (242). *In vivo* experiments on rats treated with MMC showed significant pulmonary vascular resistance, right ventricular hypertrophy, vascular remodeling, and EC proliferation within the capillary bed, accompanied by a reduction in GCN2 (242).

Research has also indicated an increased susceptibility of PAH cells to mutagens such as etoposide, bleomycin, and hydroxyurea (241, 243). Even though chromosomal abnormalities are prevalent in PAH PAECs, examination of endothelial colony-forming cells showed that their genomes remained stable through up to 15 passages. This suggests there may be no significant defects in DNA repair mechanisms and hints at potential enhancements in PAH cells (244). Most studies on DNA repair have focused on PASMCs, with limited information on PAECs.

The peroxisome proliferator-activated receptor γ (PPAR-γ), a nuclear receptor responsible for regulating fatty acid storage and glucose metabolism, has been linked to various diseases, including PH under hypoxia (245). In both PAECs and PASMCs, PPAR-γ supports cell survival and suppresses proliferation through its interaction with Apelin (246). Li et al. showed that PPAR-γ interacts with the MRN complex to facilitate ATM signaling, and it plays a crucial role in UBR5 activity, which targets ATMIN (247). Dysfunction in this pathway in PAH-PAECs leads to reduced PPAR-γ-UBR5 interaction, increased ATMIN, progressive DNA damage, and impaired repair (247).

A study by Meloche et al. reported decreased levels of microRNA miR-223 and increased Poly [ADP-ribose] polymerase 1 (PARP-1) expression in PAH, causing an imbalance in proliferation and apoptosis (248). PARP-1 is a key protein in detecting DNA damage. Treatment with PARP-1 inhibitor ABT-888 increased DNA damage in PASMCs but also induced anti-proliferative and pro-apoptotic signaling by reversing miR-204-dependent NFAT and Hif1-α levels (128). This was confirmed *in vivo*, where ABT-888 treatment reduced pulmonary artery pressure and right ventricular hypertrophy in PAH models (128, 248).

Another study by Bourgeois et al. demonstrated elevated levels of CHK-1 in PAH-PASMCs and distal pulmonary arteries, correlated with increased DNA damage markers like γH2AX and RPA32. This elevation in CHK-1 was linked to an increase in its upstream activator, phospho-ATK, and a decrease in miR-424, which increased CHK-1 levels, resulting in anti-proliferative and pro-apoptotic effects (24, 249, 250). *In vitro* experiments with the CHK1 kinase inhibitor, MK-8776, exacerbated DNA damage while controlling proliferation and promoting apoptosis (251).

Lampron et al. investigated the role of PIM1, a regulator of the non-homologous end joining (NHEJ) repair pathway, in PAH. They found increased PIM1 expression in PAH lungs and PASMCs. Inhibition of PIM1 did not increase DNA damage but reduced KU70 expression, crucial for stabilizing double-strand break ends, thereby impairing DNA repair (252). PIM1 inhibitors improved hemodynamics, reduced vascular remodeling, and enhanced apoptosis in PAH models without additional genetic insults (252).

Studies also highlighted the role of H2AX in the DNA damage response. Wang et al. found elevated EYA3 protein levels in PAH-PASMCs, suggesting increased repair mechanisms in PAH (253). EYA3 dephosphorylates H2AX, enabling repair complex assembly. Inhibition of EYA3 improved pulmonary hemodynamics and vascular remodeling in PAH models (253). These findings underscore the importance of DNA repair mechanisms in PAH pathogenesis and potential therapeutic targets. Understanding the interplay between DNA damage, repair, and epigenetics offers insight into potential therapeutic approaches for managing PAH.

### Current PAH therapies and epigenetics implications

**Figure 4:**
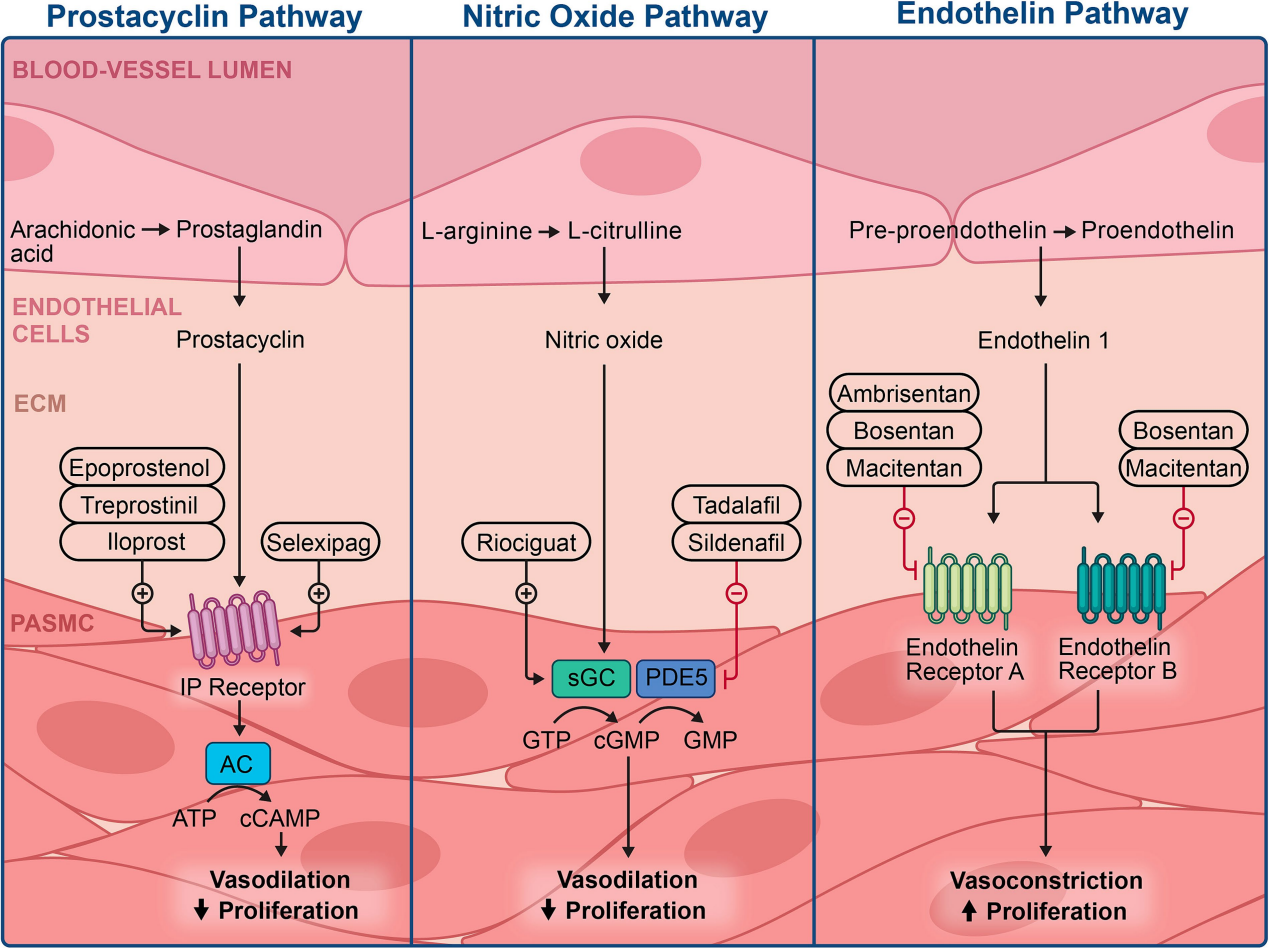
Illustration of the current therapies available in managing pulmonary hypertension and the specific mechanisms of action.

Considering the heightened severity of PAH due to the interaction of intricate genetic and epigenetic factors, identifying novel therapeutic avenues by investigating the epigenetic mechanisms involved in PAH pathogenesis is of growing interest (86). Over the years, studies have highlighted the role of epigenetic modifications, such as altered DNA methylation and histone modification, in the pathogenesis of PAH (7–9). Most research on the epigenetics of PAH has been conducted *in vivo*, necessitating further studies to understand their implications in the clinical management of PAH. Current therapies for PAH target the imbalance between vasoactive and vasodilatory mediators in PAEC, aiming to mediate vasoconstriction through vasodilatory properties. These therapies target three key pathways and are classified into four classes: endothelin receptor antagonists, phosphodiesterase type 5 inhibitors, soluble guanylate cyclase stimulators, and prostacyclin analogs. These therapies are summarized in Figure 4, and some of are believed to modulate epigenetic modifications observed in PAH.

#### Endothelial nitric oxide synthase pathway

Endothelial cells regulate pulmonary vascular tone by producing and releasing nitric oxide (NO), a potent vasodilator and key regulator of vascular homeostasis. Evidence of reduced NO production has been the rationale for using phosphodiesterase type 5 inhibitors and soluble guanylate cyclase stimulators in treating PAH (254, 255). Sildenafil, a selective and potent phosphodiesterase 5 inhibitor, can relax distal pulmonary arterioles via the classic iNO/cGMP/PKG pathway, addressing PH (256–259).

Reactive oxygen species (ROS), including free radicals like superoxide (O2-), hydrogen peroxide (H2O2), and hydroxyl anion (OH-), along with reactive nitrogen species, such as nitric oxide (NO) and peroxynitrite (ONOO-), are biologically important oxygen derivatives. They play crucial roles in vascular biology due to their oxidation/reduction potential (260, 261). All vascular cell types, including endothelial cells, smooth muscle cells, and adventitial fibroblasts, generate ROS, mainly via cell membrane-associated nicotinamide adenine dinucleotide phosphate oxidase. ROS regulates vascular function by modulating cell growth, apoptosis, migration, inflammation, secretion, and extracellular matrix protein production (260). ROS act as important intracellular and intercellular second messengers in regulating various downstream signaling pathways via reactions with protein residues (260–262).

Epigenetic changes, particularly histone modification, play a role in ROS-mediated epigenetic changes. Abnormal epigenetic alterations in the pathogenesis of PAH showed no mutation in the SOD2 gene. However, tissue-specific deficiency of SOD2 induced by methylation has been observed to increase proliferation and decrease apoptosis in PASMC, consequently disrupting redox signaling. Conversely, increasing SOD levels has been shown to ameliorate experimental PAH, highlighting the therapeutic potential of targeting epigenetic modifications (8). Epigenetic suppression of the superoxide dismutase (SOD)-2 gene through DNA methylation compromises cellular antioxidant pathways and activates hypoxia-inducible factor (HIF)-1α, contributing to mitochondrial dysfunction (263).

The impact of sildenafil on PH was assessed *in vitro*, revealing alterations in peroxisome proliferator-activated receptor γ (PPARγ), transient receptor potential canonical (TRPC)1, TRPC6, and Ki67 expression levels in hypoxic conditions in neonatal rats. PPARγ, a key transcription factor influencing BMP/TGF signaling, is highly expressed in cell types within the pulmonary vascular wall, including vascular endothelial cells and smooth muscle cells (264). It plays a crucial role in regulating lung and alveolar development, maintaining pulmonary vascular tone, and reducing PASMC proliferation, thereby preventing vascular remodeling. Research has demonstrated that PPARγ can inhibit smooth muscle cell proliferation and migration by suppressing the expression of platelet-derived growth factor expression (265, 266).

In a study, sildenafil was observed to decrease pulmonary vasoconstriction by activating PPARγ and reducing the expression of TRPC1 and TRPC6. This mechanism contributed to the reduction of pulmonary hypertension and the prevention of the thickening in the distal pulmonary arteriole wall (267). This intervention also reversed hypoxia induced elevation in right ventricular mean pressure and right ventricular hypertrophy index, reduced pulmonary arterial remodeling, and inhibited PASMC proliferation in neonatal rats exposed to hypoxia (264). Sonneveld et al. suggested that sildenafil-induced increases in cGMP levels lead to the activation of PKG-1 and, subsequently, PPARγ (259). Sildenafil’s inhibition of TRPC expression and PASMC proliferation were diminished by the PPARγ inhibitor GW9662 and PPARγ small interfering RNA (264, 265). Although sildenafil is believed to modulate the epigenetic regulation of the PPARγ pathway, the exact mechanism remains unclear (265, 266).

#### BMP signaling pathway

The bone morphogenetic protein (BMPs) signaling pathway is another important pathway implicated in the PAH pathogenesis BMPs belong to the transforming growth factor-β (TGF-β) family, which consists of cytokines secreted by epithelial cells and fibroblasts (64) BMPs are essential in regulating growth, differentiation, and apoptosis in various cell types, including pulmonary vascular endothelium and fibroblasts (65).

BMPR-II is highly expressed in PAEC and, to a lesser extent, in PASMC and fibroblasts (267). However, regardless of the presence of BMPR-II mutations, there is a significant reduction in BMPR-II expression in the pulmonary vasculature, a critical factor in the development of PAH.

Dysfunction of BMPR-II is well documented in the pathogenesis of PAH, with 14–42% of individuals with known BMPR-II mutations developing detectable PAH in their lifetime (268–270). Heritable PAH (HPAH), a subtype of PAH, exhibits germline mutations in the BMPR-II gene in 70–80% of cases (270, 271). Additionally, 11–40% of IPAH patients without a family history also harbor BMPR-II mutations, highlighting the significant role of BMP signaling in PAH pathogenesis (272, 273).

Additionally, mutations related to PAH have been identified in other TGF-β family members that functionally interact with BMPR-II in PASMCs and PAECs. Mutations in BMPR-II transcriptional mediators, such as SMAD1, SMAD4, SMAD5, SMAD8, and the scaffolding protein caveolin-1, predispose individuals to PAH (274–276). In rare cases, HPAH patients especially those with hereditary hemorrhagic telangiectasia, exhibit germline mutations in activin receptor-like kinase type 1 (ACVRL1 or ALK1) or endoglin genes (74). Other genes associated with PAH include EIF2AK4, BMP10, ENG, KCNK3, ABCC8, AQP1, CAV1, TBX4, GDF2, SOX17, G6PD, KDR, and ATP13A3 (277–279). These findings indicate a complex interplay of genetic, environmental, and epigenetic factors resulting in varied phenotypic expressions.

Within the TGF-β family, ligands bind to constitutively active TGFβ type II serine/threonine kinase receptors (TβRII), forming stable receptor complexes that activate downstream signals (280). The ligand specificity for distinct receptor complexes is crucial for the tissue-specific nature of BMP signaling (281, 282). BMPs form complexes with TβRII, leading to phosphorylation and recruitment of TGFβ type I serine/threonine kinase receptors (TβRI). Activated type I receptors phosphorylate cytoplasmic signaling proteins known as Smads, facilitating TGF-β superfamily signal transduction (69, 280, 283–285).

**Figure 5:**
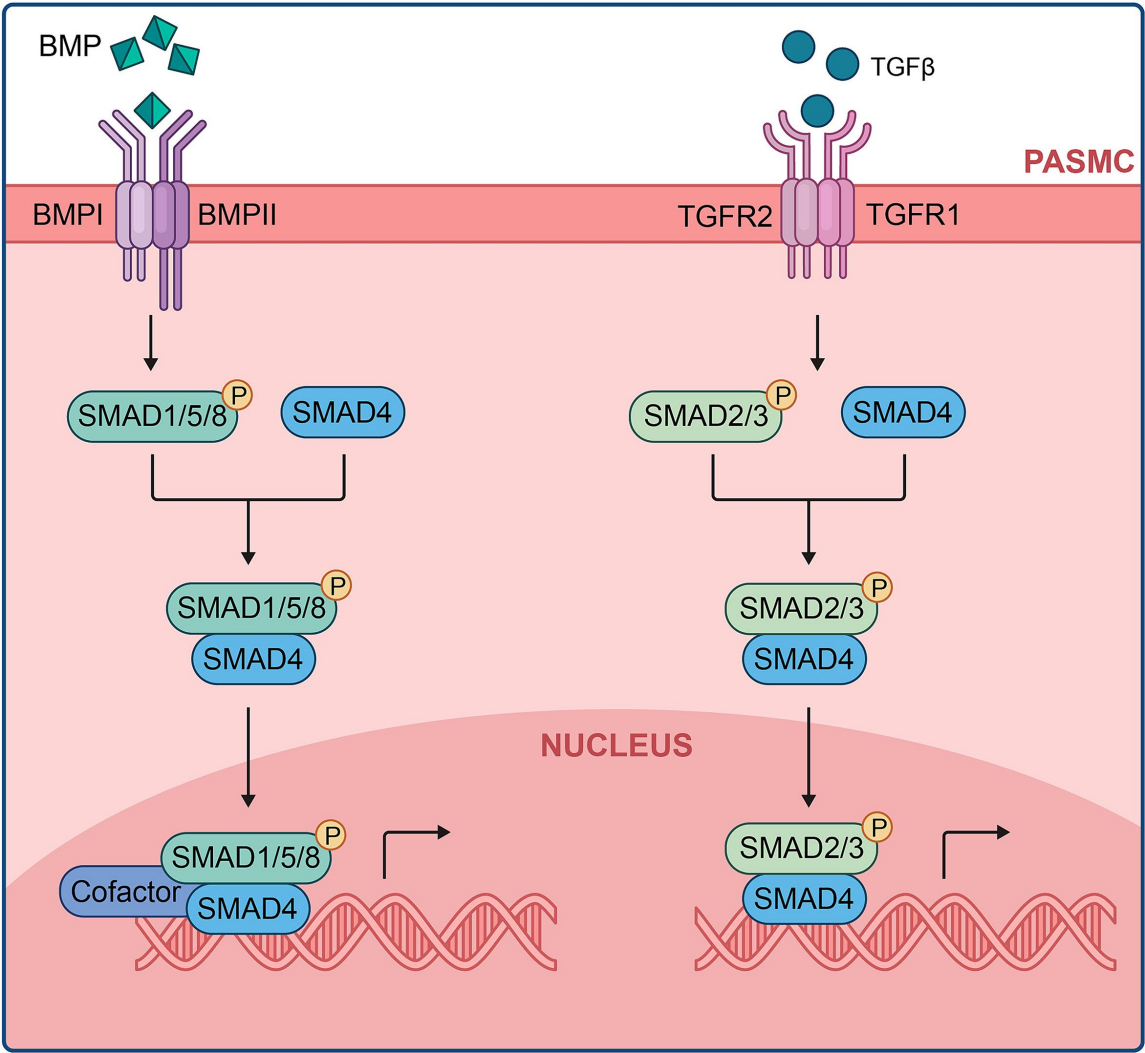
BMP/TGF-β signaling cascade at the cellular level.

The Smad signaling cascade begins with phosphorylated TβRI binding to receptor-mediated Smads (R-Smads), specifically Smads-1, −5, and-8, forming complexes with the co-Smad, Smad4. Phosphorylated Smads, having a higher affinity for Smad-4, translocate to the nucleus (Figure 5). BMP9, identified as a BMP ligand, signals by binding to the endothelial receptor BMPR-II and ALK1, along with the co-receptor endoglin. This signaling pathway is crucial for maintaining pulmonary vascular integrity (73). This mechanism also explains the rare occurrence of severe PAH in families with hereditary hemorrhagic telangiectasia due to ALK-1 mutations (74).

BMP/Smad signaling disruption by BMPR-II mutation is heterogeneous and mutation-specific (286). Missense mutations involving cysteine substitution within the ligand-binding or kinase domain of BMPR-II reduce the trafficking of the mutant protein to the cell surface (287). The activity of the BMPR-II pathway depends on the specific BMPR-II ligands for each vascular cell type. BMP9 and BMP10 primarily influence PAECs in pulmonary arteries (288), while BMP2 and BMP7 facilitate PASMC apoptosis, and BMP4 promotes SMC proliferation (289–291).

A decrease in BMPR-II in PASMCs may diminish BMP2/4/7 signaling, leading to the accumulation of hyperproliferative SMCs that are resistant to apoptosis, which is characteristic of muscularization in the distal arterial of PAH patients. Non-cysteine mutations within the kinase domain allow the protein to reach the cell surface but do not activate Smad-responsive luciferase reporter genes. Additionally, many mutations result in the degradation of the mutant transcript via nonsense-mediated mRNA decay, resulting in haploinsufficiency (292, 293).

Researchers have proposed that chronic loss of BMPR-II involves epigenetic modifications of the promoter regions of genes (294). Soon et al. found that the loss of SOD3 expression in Bmpr2+/− cells was reversed by treating the cells with the histone deacetylase inhibitor trichostatin A. Various microRNAs, histone deacetylases, and abnormal DNA methylation modifications have been linked to PAH, with some specifically implicated in the downregulation of BMPR-II (8, 295, 296).

Various other mechanisms of the BMPR-II signaling pathway have emerged as promising targets for new PAH therapies. One consequence of BMPR-II insufficiency is the shift from downstream SMAD1/5/8 activation to SMAD2/3/4 activation via the activin receptor type IIA (ACTRIIA), reducing BMPR-II/SMAD1/5/8 antiproliferative signaling and promoting pulmonary vascular remodeling through increased ACTRIIA/SMAD2/3 signaling (297). The recently FDA-approved PAH therapy Sotatercept, consisting of a fusion protein combining activin receptor type IIA with the Fc domain of human IgG1, operates by binding free activins, the ligands for ACTRIIA/B, thereby rebalancing SMAD signaling and promoting antiproliferation.

Although a recognized connection exists between BMPR-II mutations and pathological epigenetic modifications, further research is needed to determine the role of sotatercept and the mechanisms of epigenetic modification in these complex signaling pathways (298).

### Current research gaps

The complexity of PAH etiopathogenesis cannot be overstated, with its disease state involving multifactorial pathogenesis contributing to its variable expression. In recent years, growing attention has been directed toward the role of epigenetic mechanisms in PAH through both experimental and clinical studies. These investigations illuminate pathogenic mechanisms and uncover potential therapeutic and curative targets.

Epigenetic modifications are emerging as a critical factor in the pathogenesis of PAH, integrating various risk factors, including environmental exposures and modulating genetic expression (114). Despite significant progress in understanding epigenetics, the identification of clinically therapeutic epigenetic treatments remains challenging. Most of the identified epigenetic therapeutic targets have been tested in animal models.

Further research is crucial to enhance our understanding of epigenetic mechanisms, as robust data suggest that epigenetic modulators hold great promise as emerging targets for PAH therapy. Additionally, ongoing research will contribute to a better understanding of the safety profile of new treatments and their impact on other gene targets.

## Author contributions

CE: Conceptualization, Investigation, Resources, Supervision, Validation, Writing – original draft, Writing – review & editing. ZS: Writing – original draft, Writing – review & editing.
